# Mining the Plasma Cell Transcriptome for Novel Cell Surface Proteins

**DOI:** 10.3390/ijms19082161

**Published:** 2018-07-24

**Authors:** Stephanie Trezise, Alexander Karnowski, Pasquale L. Fedele, Sridurga Mithraprabhu, Yang Liao, Kathy D’Costa, Andrew J. Kueh, Matthew P. Hardy, Catherine M. Owczarek, Marco J. Herold, Andrew Spencer, Wei Shi, Simon N. Willis, Stephen L. Nutt, Lynn M. Corcoran

**Affiliations:** 1 The Walter and Eliza Hall Institute of Medical Research, Parkville, VIC 3052, Australia; trezise.s@wehi.edu.au (S.T.); fedele.p@wehi.edu.au (P.L.F.); liao@wehi.edu.au (Y.L.); dcostakj@gmail.com (K.D.); kueh@wehi.edu.au (A.J.K.); herold@wehi.edu.au (M.J.H.); shi@wehi.edu.au (W.S.); willis@wehi.edu.au (S.N.W.); corcoran@wehi.edu.au (L.M.C.); 2 Department of Medical Biology, University of Melbourne, Parkville, VIC 3010 Australia; 3 CSL Limited, Parkville, VIC 3010, Australia; alexander.karnowski@csl.com.au (A.K.); Matt.Hardy@csl.com.au (M.P.H.); Catherine.Owczarek@csl.com.au (C.M.O.); 4 Haematology Department, Monash Health, Clayton, VIC 3168, Australia; 5 Department of Clinical Haematology, Alfred Health, Melbourne 3004, Australia; durga.mithraprabhu@monash.edu (S.M.); aspencer@netspace.net.au (A.S.); 6 Australian Centre for Blood Diseases, Monash University, Melbourne 3004, Australia; 7 Department of Computing and Information Systems, University of Melbourne, Parkville, VIC 3010, Australia

**Keywords:** plasma cell, antibody, CLPTM1L, ITM2C, PLPP5, membrane protein

## Abstract

Antibody Secreting Cells (ASCs) are a fundamental component of humoral immunity, however, deregulated or excessive antibody production contributes to the pathology of autoimmune diseases, while transformation of ASCs results in the malignancy Multiple Myeloma (MM). Despite substantial recent improvements in treating these conditions, there is as yet no widely used ASC-specific therapeutic approach, highlighting a critical need to identify novel methods of targeting normal and malignant ASCs. Surface molecules specifically expressed by the target cell population represent ideal candidates for a monoclonal antibody-based therapy. By interrogating the ASC gene signature that we previously defined we identified three surface proteins, Plpp5, Clptm1l and Itm2c, which represent potential targets for novel MM treatments. *Plpp5*, *Clptm1l* and *Itm2c* are highly and selectively expressed by mouse and human ASCs as well as MM cells. To investigate the function of these proteins within the humoral immune system we have generated three novel mouse strains, each carrying a loss-of-function mutation in either *Plpp5*, *Clptm1l* or *Itm2c*. Through analysis of these novel strains, we have shown that Plpp5, Clptm1l and Itm2c are dispensable for the development, maturation and differentiation of B-lymphocytes, and for the production of antibodies by ASCs. As adult mice lacking either protein showed no apparent disease phenotypes, it is likely that targeting these molecules on ASCs will have minimal on-target adverse effects.

## 1. Introduction

The differentiation of mature B cells into Antibody Secreting Cells (ASCs) is an essential part of the adaptive immune response and underlies virtually all current vaccination strategies. The antibodies that these cells produce are important for the elimination of infecting pathogens and the persistent secretion of these antibodies after pathogen clearance provides long-term protection against re-infection. However, the generation of self-reactive antibodies is the driver of many autoimmune diseases, including Systemic Lupus Erythematosus and Sjörgrens Syndrome and we currently lack effective methods of targeting the long-lived ASC population [1]. Furthermore, Multiple Myeloma (MM) is an ASC malignancy that constitutes approximately 10% of all hematological malignancies [2]. MM derives from the clonal expansion of a transformed post germinal center plasma cell, leading to bone marrow plasmacytosis and production of a monoclonal immunoglobulin. In addition to the consequences of effacement of normal bone marrow hematopoiesis resulting in anemia and other cytopenias, patients with MM develop specific end-organ sequale including renal failure, hypercalcemia and bone lesions [3]. Despite recent advances in treatment for this disease, through the use of proteasome inhibitors and immunomodulatory drugs, MM remains incurable and the median survival from diagnosis is only 5–6 years [2]. Recently, monoclonal antibody (mAb)-based therapies have been at the forefront of novel treatments for MM.

mAb-based immunotherapies are a highly specific and effective method of targeting a given cell type for depletion. The power of this therapy in the treatment of hematological cancers was first demonstrated by Rituximab, directed against CD20, a cell surface protein that is expressed on mature B cells, however, Rituximab is ineffective against MM and ASCs, as they no longer express CD20 [4,5]. There are several mAb therapies targeting MM cells that are currently in clinical use including Elotuzumab, and Daratumumab, which target Signaling lymphocytic activation molecule F7 (SLAMF7) [6,7] and CD38 [8], respectively. Unfortunately, the development of clones resistant to these therapies ultimately means that cancer regrowth is inevitable. Developing new targeted therapies for MM to use in combination with existing ones increases the likelihood that all clones could be targeted during treatment and would allow for more complete clearance of the cancer.

To increase our understanding of ASC biology and to enable us to identify novel therapeutic strategies to target pathogenic ASC, including MM, we have previously examined the transcriptional changes that occur during B cell differentiation in the mouse [9]. This study allowed us to define a robust ASC gene signature, a collection of genes consistently upregulated during the process of terminal differentiation. It is highly likely that this signature contains surface molecules, which are selectively expressed on ASCs and will consequently be suitable targets for an ASC directed immunotherapy. In agreement with this conclusion, all the current targets of mAb therapy for MM are found within the ASC gene signature, however, there is also a large collection of genes whose function remains either undetermined or that had not previously been associated with ASCs. We believe that some of these genes will present potential novel ways of targeting ASCs and MM.

We have identified three genes within the ASC gene signature, phospholipid phosphatase 5 (*Plpp5*), cleft-lip and palate transmembrane protein 1-like (*Clptm1l*) and integral membrane protein type 2 C (*Itm2c*), that are candidates for a mAb-based therapy to target pathogenic plasma cells. All three proteins display surface expression, and their highly conserved human homologues are expressed in both healthy ASCs and in MM cells. To identify possible side-effects of targeting these three proteins, we generated three novel stains of mice, each carrying a loss-of-function mutation in one of the three candidate genes. This work details the first analysis of *Plpp5*, *Clptm1l* or *Itm2c* function within immune cells.

## 2. Results

### 2.1. Identification of Candidate Cell Surface Proteins in Anibody Secreting Cells

**Figure 1:**
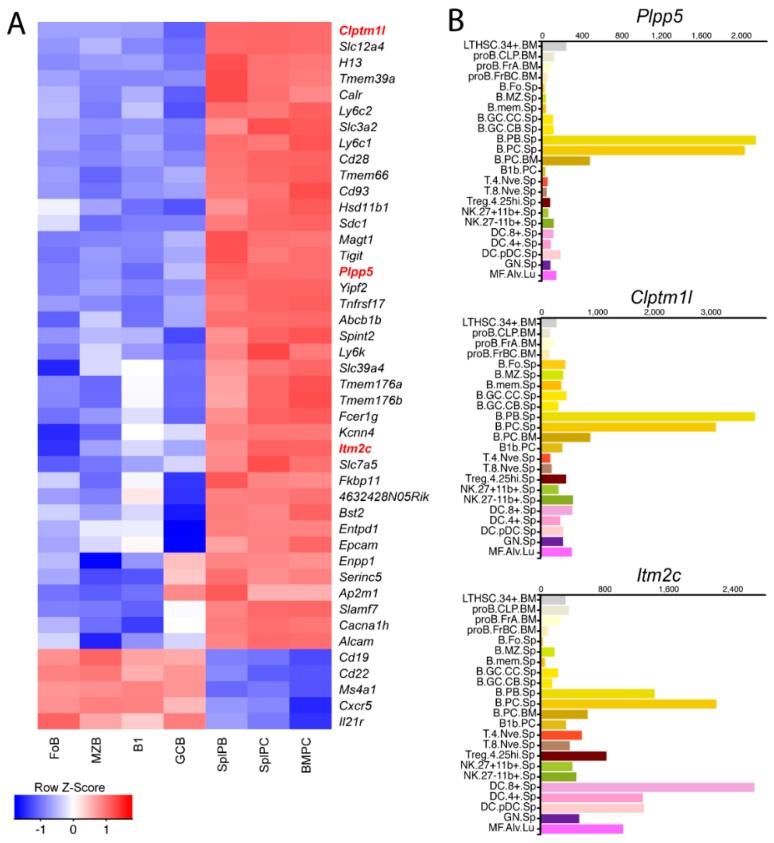
Identification of genes encoding novel surface proteins in mouse ASCs. (**A**) Expression profiles of genes within the ASC gene signature that encode transmembrane proteins that are either known or predicted to be expressed on the plasma membrane. The expression of five additional genes encoding cell surface proteins expressed in B cells, but not plasma cells is shown for comparison. The positions of *Plpp5*, *Clptm1l* and *Itm2c* are highlighted in red. Expression is represented as a Z-score as defined by the legend; (**B**) expression of *Plpp5*, *Clptm1l* and *Itm2c* in selected mouse immune cell populations. Data obtained from the Immgen Consortium. Expression value normalized by DEseq2. Immgen nomenclature: BM, bone marrow; Sp, splenic; PC, peritoneal cavity; Lu, lung; LTHSC.34^+^, CD34^+^ long-term hematopoietic stem cell; proB.CLP, common lymphoid progenitor; proB.FrA, pre-pro-B cell; proB.FrBC, pro-B cell; B.Fo, Follicular B cell; B.MZ, MZ B cell; B.mem, memory B cell; B.GC.CC, GC centrocyte; B.GC.CB, GC centroblast; B.PB., Plasmablast; B.PC, Plasma cell; T.4.Nve, naïve CD4^+^ T cell; T.8.Nve, naïve CD8^+^ T cell; Treg.4.25^hi^, CD25^hi^ Treg; NK.27^+^11b^−^, CD27^+^ Cd11b^−^ NK cell; DC.8^+^, CD8^+^ Dendritic Cell (DC); DC.4^+^, CD4^+^ DC; DC.pDC, plasmacytoid DC; GN, neutrophil; MF.Alv, alveolar macrophage.

We have previously generated gene expression profiles for mature B cells and ASC populations and identified a subset of genes, termed the ASC gene signature, which are upregulated during the process of B cell terminal differentiation [9]. From this signature, we searched the current literature for proteins with evidence of surface localization, resulting in a shortened list of 39 genes encoding membrane spanning proteins for which there is some evidence for cell surface localization (Figure 1A). In addition to the established markers of plasma cells, including *Sdc1* (*Cd138*) and *Slamf7*, there were many genes with no known association with ASC biology, thus representing potential novel targets for an ASC specific therapy. From this shortened list we selected three genes for further investigation, *Plpp5*, *Clptm1l* and *Itm2c*. All three of these genes are poorly characterized and currently have no published association with any immune cell type. We next interrogated the Immgen Consortium database (www.immgen.org) to examine the expression of these three genes across a range of immune cell populations (Figure 1B) [10]. *Plpp5* and *Clptm1l* displayed high expression almost exclusively in ASC populations, while *Itm2c* was also highly expressed in dendritic cells. The selective expression of these genes suggests that they are candidates for a possible ASC-specific therapy.

### 2.2. Plpp5, Clptm1l and Itm2c Are Highly Conserved between Mice and Humans

**Figure 2:**
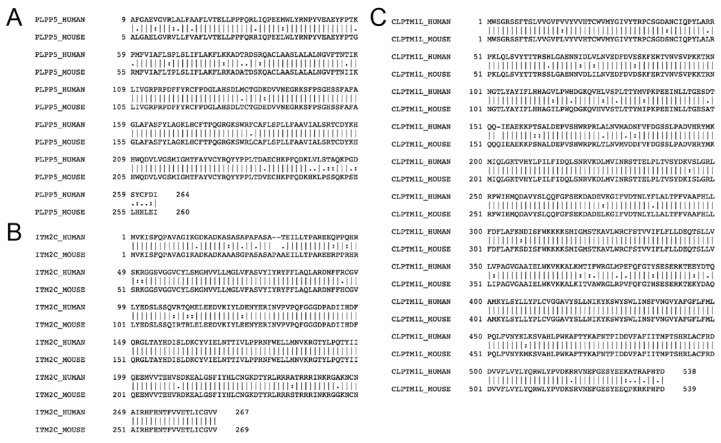

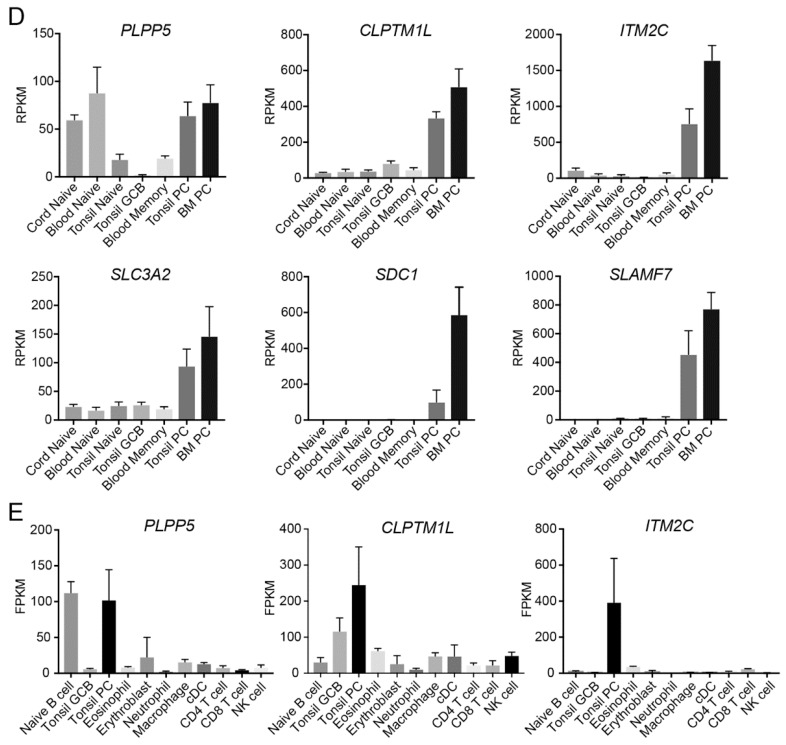
Expression of the human homologues in ASCs. Alignment of mouse and human amino acid sequences for (**A**) PLPP5, (**B**) ITM2C, and (**C**) CLPTM1L. Symbols indicate conserved (I), highly similar (:), and similar (.) residues. (**D**) RNAseq showing the expression of *PLPP5*, *CLPTM1L*, *ITM2C* and three established clinical candidates *SLC3A2*, *SDC1* and *SLAMF7* in purified human B cell and ASC populations. Data are the mean reads per kilobase per million reads (RPKM) ± SD from 3–5 donors each. Cord naïve B cell (CD19^+^ CD20^+^ HLA-DR^+^ IgG^−^), blood naïve B cell (CD19^+^ IgD^+^ CD27^−^), tonsil naïve B cell (CD19^+^ CD38^−^ CD27^−^), tonsil germinal center (GCB) B cell (CD19^+^ CD38^+^ CD27^+^), blood memory B cell (CD19^+^ IgD^−^ CD27^+^ CD38^−/low^), tonsil plasma cell/plasmablast (PC, CD19^+^ CD27^++^ CD38^++^), bone marrow plasma cell (BM PC, CD138^++^ CD38^+^). (**E**) RNAseq showing the expression of *PLPP5*, *CLPTM1L* and *ITM2C* in human immune cell populations. Data obtained from the BLUEPRINT consortium (www.blueprint-epigenome.eu). Data are the mean fragments per kilobase per million reads (FPKM) ± SD. Blood naïve B cell (*n* = 5, CD19^+^ CD20^+^ CD23^+^ CD38^low^), tonsil GCB cell (*n* = 3, CD19^+^ CD20^+^ CD38^medium^), tonsil PC (*n* = 21, CD20^medium^ CD38^++^), blood eosinophil (*n* = 2, CD66^+^ CD16^−^), blood erythroblast (*n* = 8, CD36^+^ CD71^+^ CD235a^+^), blood neutrophil (*n* = 16, CD66b^+^ CD16^+^), cultured macrophages (*n* = 18) and conventional dendritic cell cDC (*n* = 3), blood CD4^+^ T cell (*n* = 10, CD3^+^ CD4^+^ CD45RA^+^), blood CD8^+^ T cell (*n* = 2, CD3^+^ CD8^+^ CD45RA^+^), blood natural killer (NK) cell (*n* = 4, CD3^−^ CD56^+^ CD16^dim^).

Having identified Plpp5, Clptm1l and Itm2c as candidate ASC markers in the mouse, we next examined whether their sequences and expression patterns were conserved in humans. We performed pairwise sequence analysis of the mouse and human amino acid sequences for each of PLPP5, CLPTM1L and ITM2C, and found that they have sequence identity of 87.9%, 92.8%, and 92.9% respectively (Figure 2A–C). To determine whether *PLPP5*, *CLPTM1L* and *ITM2C* have similar expression patterns in mice and humans, we examined the expression of each gene in human B cell and ASC populations (Figure 2D). The pattern of expression of *CLPTM1L* and *ITM2C* during the terminal differentiation of both mouse and human B cells was very similar; low expression in B cell subsets, which increased markedly in ASC populations. *CLPTM1L* and *ITM2C* displayed the same pattern of expression as *SLC3A2*, *SDC1* and *SLAMF7*, which are current targets of ASC-directed immunotherapies. The expression of *PLPP5* differed between mice and humans, with expression in both naïve B cells and ASCs in humans while expression in mice was exclusive to ASCs. To determine whether the expression of these genes within human immune cell populations mirrored expression in the mouse we interrogated the BLUEPRINT consortium RNAseq database (http://www.blueprint-epigenome.eu) and observed that *PLPP5*, *CLPTM1L* and *ITM2C* expression was similarly restricted to B cells and ASCs (Figure 2E) [11]. The high degree of sequence identity and similar expression patterns suggests that it is likely that these genes serve a similar function in both mice and humans ASCs.

### 2.3. Confirmation of Plpp5, Clptm1l and Itm2c Surface Expression and Prediction of Membrane Topology

**Figure 3:**
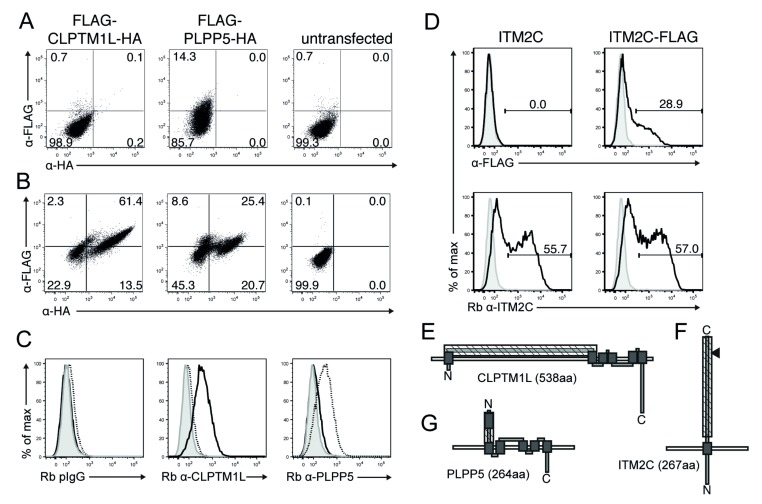
Cell surface expression of human PLPP5, CLPTM1L and ITM2C. FS-293F cells were transiently transfected with human CLPTM1L or PLPP5 expression constructs with N-terminal FLAG tags and C-terminal HA tags. (**A**) Cell surface or (**B**) Intra-cellular flow cytometric staining with anti-FLAG and anti-HA antibodies; (**C**) cell surface staining of transduced FS-293F cells with Rabbit (Rb) IgG control, Rb anti-CLPTM1L polyclonal (p)Ab and Rb anti-PLPP5 pAb (grey histograms: un-transfected cells, black lines: FLAG-Human-CLPTM1L-HA cells, dashed line: FLAG-Human-PLPP5-HA cells); (**D**) FS-293F cells were transiently transfected with a human ITM2C expression constructs with or without C-terminal FLAG tags. Flow cytometric analysis of surface anti-FLAG and Rb anti-ITM2C pAb staining (grey histograms: un-transfected cells, black lines: Human-ITM2C transfected cells); (**E**–**G**) predicted domain structure and cell surface localization of (**E**) CLPTM1L, (**F**) ITM2C and (**G**) PLPP5. (dark-grey boxes: putative TM-domains, hatched boxes: peptides used for pAb generation, black triangle Furin cleavage site).

Since there has been only limited analysis of the protein products of *PLPP5*, *CLPTM1L* and *ITM2C* we conducted an in silico analysis with several tools (Phobius, Spoctopus, TMHMM2 and TMMOD [12,13,14,15] to predict putative signal peptides, trans-membrane (TM) domains and membrane topology. The majority of the tools predicted murine and human CLPTM1L and PLPP5 being type IIIb membrane proteins, lacking signal peptides with six putative TM domains. Murine and human ITM2C was predicted to be a type II transmembrane protein without a signal peptide and one putative TM domain. To confirm surface expression and orientation we generated amino- and/or carboxy-terminal tagged expression constructs for each protein. Flow cytometric analysis of transiently expressed CLPTM1L and PLPP5, using tag-specific antibodies showed only surface staining of the N-terminal FLAG tag of PLPP5 (Figure 3A). However, both tags were detected in an intra-cellular stain (Figure 3B). Using anti-CLPTM1L or PLPP5 specific polyclonal antibodies that were raised against peptides localized within the central to N-terminal region, we detected surface expression of both transmembrane proteins (Figure 3C).

Carboxy-terminal-tagged ITM2C was detected on the cell surface of transiently transfected FS-293F cells by surface staining with a tag-specific antibody and an ITM2C specific antibody (Figure 3D). However, we observed about 30% of tag-positive cells in the tag-specific surface stain, but almost 60% of ITM2C positive cells using the specific antibody. This discrepancy is likely due to protein cleavage of the carboxy-terminal end of ITM2C [16]. The surface staining of human CLPTM1L and ITM2C fits well with the predicted topology (Figure 3E,F) and a previous study [17], demonstrating that these proteins are expressed on the cell surface. The cell surface detection of the N-terminal tag of PLPP5 suggests that the predicted trans-membrane domain at the N-terminal end is not utilized and that a 57 amino acid N-terminal region that contains the polyclonal antibody-binding site is exposed at the cell surface (Figure 3G).

### 2.4. PLPP5, CLPTM1L and ITM2C Are Highly Expressed in Multiple Myeloma

**Figure 4:**
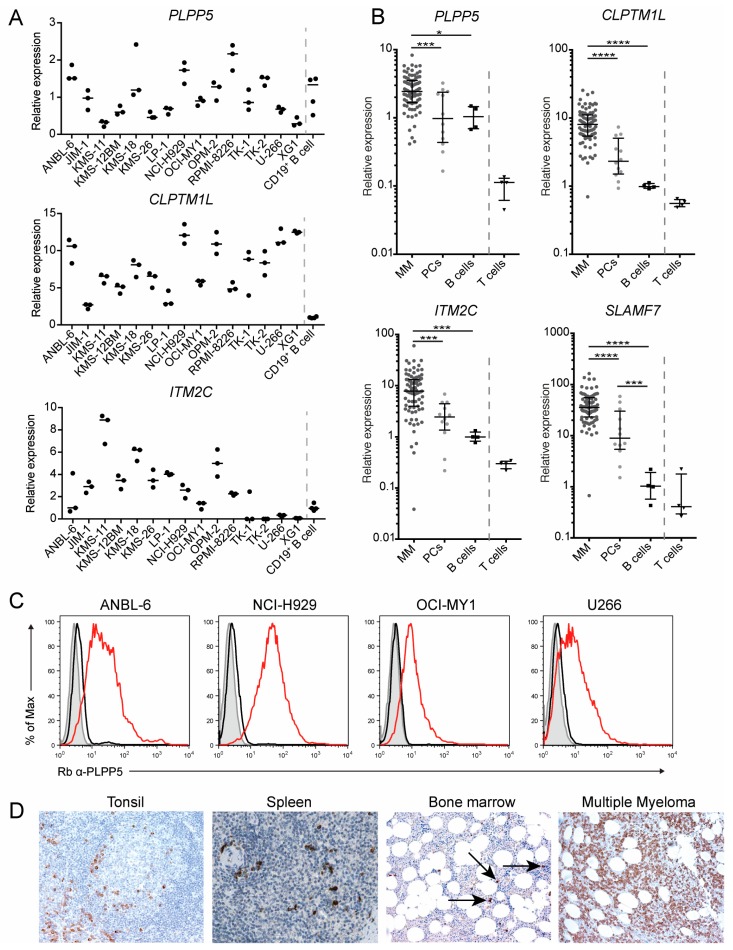
*PLPP5*, *CLPTM1L* and *ITM2C* are expressed in human Multiple Myeloma. (**A**) Expression of *PLPP5*, *CLPTM1L*, and *ITM2C* in human myeloma cell lines and primary human CD19^+^ B cells as determined by qPCR. Expression data was normalized by the house-keeping genes *GAPDH*, *GPBP1* and *TPT1*. Bars show median normalized expression of experimental replicates (*n* = 3 myeloma cell lines, *n* = 4 for CD19^+^ primary peripheral B cells); (**B**) expression of *PLPP5*, *CLPTM1L, ITM2C* and *SLAMF7* in primary bone marrow MM samples (*n* = 83), sorted plasma cells (*n* = 12), peripheral blood B cells (*n* = 4) and T cells (*n* = 4). Expression data was normalized as in (**A**). Bars show median expression and interquartile range. One-way ANOVA with Tukey’s multiple comparison test on log transformed data from MM, plasma cells and B cells; * (*p* < 0.05), *** (*p* < 0.001), **** (*p* < 0.0001). (**C**) cell surface expression of PLPP5 in human myeloma cell lines was determined by flow cytometry. Grey histograms: unstained cells, black lines: anti-rabbit (Rb) IgG control, red lines: Rb anti-PLPP5; (**D**) expression of CLPTM1L in human tonsil, spleen and bone marrow trephine from patients with non-Hodgkin’s Lymphoma (labeled, Bone marrow) or multiple myeloma. Spleen image was obtained from the Human Protein Atlas v18 (www.proteinatlas.org/ENSG00000049656-CLPTM1L/tissue/spleen#img). Images were captured at 100× magnification.

To determine whether PLPP5, CLPTM1L and ITM2C were potential targets in MM cells, we first analyzed the expression of each gene in a range of MM cells lines using qPCR (Figure 4A). The expression of *PLPP5*, *CLPTM1L* and *ITM2C* varied between different established human myeloma cell lines, however, all cell lines examined had detectable expression of at least one of these genes of interest. We next determined the expression of *PLPP5*, *CLPTM1L*, *ITM2C* and *SLAMF7* (as a positive control) in primary MM and plasma cell (PC) samples (Figure 4B). All four genes showed significantly increased expression in MM samples compared to PCs. To confirm that there was a corresponding increase in protein expression we examined the expression of PLPP5 in a selection of MM cell lines (Figure 4C). In agreement with our qPCR data, all examined cell lines showed cell surface expression of PLPP5.

Finally, we used a commercially available anti-CLPTM1L antibody to perform immunohistochemistry on human tonsil and on bone marrow from patients with either non-Hodgkin’s lymphoma (non-involved bone marrow) or MM (Figure 4D). Staining revealed that there were a small number of CLPTM1L-positive cells in the bone marrow and in the tonsil surrounding a follicle, which was the expected location and prevalence for ASCs. Our staining was in agreement with images obtained from the Human Protein Atlas [18], which showed a small number of anti-CLPTM1L-labeled cells in human spleen. Staining of bone marrow from a patient with MM revealed widespread CLPTM1L-positive cells, further supporting our qRNA analysis showing high *CLPTM1L* expression in MM. Together, these results confirm that *PLPP5*, *CLPTM1L* and *ITM2C* are expressed in human ASCs and MM, and are therefore candidates for ASC-directed immunotherapy.

### 2.5. Generation of Plpp5, Clptm1l and Itm2c Knock-Out Mouse Models

As noted above PLPP5, CLPTM1L and ITM2C are poorly characterized proteins, whose functions are unknown, although mutation in *CLPTM1L* has been associated with several human cancers (see Discussion). To interrogate the roles of these proteins within ASCs we generated knock-out mouse strains, each carrying loss-of-function mutations in either *Plpp5*, *Clptm1l* or *Itm2c*.

**Figure 5:**
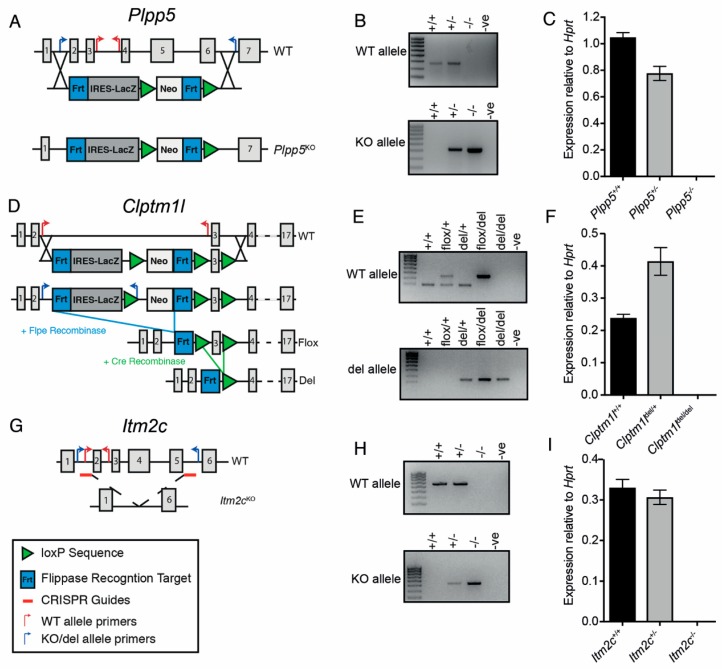
Generation of *Plpp5*, *Clptm1l* and *Itm2c* deficient mouse models**.** (**A**) Strategy for the generation of the non-functional (−) *Plpp5* allele; (**B**) gel electrophoresis of PCR products amplified from WT, *Plpp5*^+/−^ and *Plpp5*^−/−^ pups. –ve, negative control water only; (**C**) mRNA expression of *Plpp5* in WT, *Plpp5*^+/−^ and *Plpp5*^−/−^ B cell cultures following 4 days of LPS stimulation; (**D**) strategy for the generation of the conditional (flox) and non-functional (del) *Clptm1l* alleles; (**E**) gel electrophoresis of PCR products amplified from WT, *Clptm1l*^flox/+^, *Clptm1l*^del/+^, *Clptm1l*^flox/del^ and *Clptm1l*^del/del^ pups; (**F**) mRNA expression of *Clptm1l* in WT, *Clptm1l*^del/+^ and *Clptm1l*^del/del^ B cell cultures following 4 days of LPS stimulation; (**G**) strategy for the generation of the non-functional (−) *Itm2c* allele using CRISPR. The location of the small guide RNAs are indicated; (**H**) gel electrophoresis of PCR products amplified from WT, *Itm2c*^+/−^ and *Itm2c*^−/−^ pups; (**I**) mRNA expression of *Itm2c* in WT, *Itm2c*^+/−^ and *Itm2c*^−/−^ B cell cultures following 4 days of LPS stimulation. All mRNA expression was determined using qRT-PCR and normalized to *Hprt* expression. Data are the mean ± SD of technical triplicates. Genomic structures in (**A**,**D**,**G**) indicate the numbered exons of each gene (gray boxes) and the mutant alleles generated. The targeting constructs for *Plpp5* and *Clptm1l* are shown below the WT genes. The positions of genotyping primers, LoxP and Frt sites are indicated.

The *Plpp5* deficient mice were generated using embryonic stem (ES) cells obtained from the KOMP Repository (Figure 5A). The ES cells carried an IRES-LacZ cassette replacing exons 2–6 of the *Plpp5* gene. The ES cells were injected into day 3.5 blastocysts to generate chimeric mice, which were then bred to generate heterozygous and ultimately homozygous progeny. PCR amplification using primer pairs that spanned the insertion site confirmed the targeting vector was inserted correctly (Figure 5B). To demonstrate that the insertion of the targeting vector disrupted the *Plpp5* locus, we measured the abundance of *Plpp5* mRNA transcripts in lipopolysaccharide (LPS) stimulated wild type (WT), *Plpp5*^+/−^ and *Plpp5*^−/−^ B cell cultures (Figure 5C). This analysis confirmed that there was no detectable *Plpp5* mRNA in the culture from *Plpp5*^−/−^ mice.

We generated *Clptm1l* mutant mice using ES cells obtained from the EUCOMM Consortium (Figure 5D). The construction of the targeting vector meant that the inactivation of *Clptm1l* could be performed conditionally (“flox” allele) or constitutively (“del” allele). As before, the ES cells were used to generate chimeric mice. These mice were crossed to Flp recombinase-expressing mice to excise the Neo-LacZ selection cassette and produce the functional *Clptm1l^flox^* allele. *Clptm1l^flox/+^* mice were crossed to a strain expressing Cre recombinase in the germline to generate the *Clptm1l*^del^ allele. The excision of exon three creates a frame shift mutation with a stop codon immediately downstream. The correct structure of each allele was confirmed by PCR (Figure 5E). WT, *Clptm1l*^del/+^ and *Clptm1l*^del/del^ B cells were stimulated with LPS and the abundance of *Clptm1l* mRNA in each culture was measured to confirm the absence of *Clptm1l* expression in del/del mice (Figure 5F). Curiously, we observed the abundance of *Clptm1l* mRNA was greater in the *Clptm1l*^del/+^ cells than in the WT cell (Figure 5F). To investigate a potential consequence of this finding, our subsequent studies also included *Clptm1l*^del/+^ mice.

We generated an *Itm2c* null mouse de novo using CRISPR/Cas9-mediated gene deletion. Guide RNAs were designed to target intronic sequences flanking exons 2 and 5, resulting in the deletion of the intervening exons (Figure 5G). This region was amplified by PCR to confirm that the desired resection event had occurred (Figure 5H). As before, we stimulated naïve B cells from WT, *Itm2c*^+/−^ and *Itm2c*^−/−^ mice with LPS and measured the abundance of *Itm2c* mRNA in each culture (Figure 5I). *Itm2c* transcripts were not detectable in the cultures produced from *Itm2c*^−/−^ cells.

*Plpp5*^−/−^, and *Itm2c*^−/−^ mice were viable and showed no overt physical defects or disease phenotype. *Plpp5*^−/−^ and *Itm2c*^−/−^ pups were born at the expected frequencies from +/− × +/− matings, suggesting that the loss of either gene did not have a detrimental impact on viability. In contrast, *Clptm1l*^del/del^ mice were born at a significantly lower than expected frequency from heterozygous mating pairs (Table 1). Additionally, fewer than 15% of the *Clptm1l*^del/del^ pups that were born, survived past day 2 (Table 2). Curiously, those *Clptm1l*^del/del^ pups that survived the neo-natal period appeared healthy and indistinguishable from their littermates. Together, this suggests that Clptm1l has a function that is important for the survival of embryonic and neonatal mice, which warrants further investigation in the future. Both male and female *Plpp5*^−/−^, *Clptm1l*^del/del^ and *Itm2c*^−/−^ mice were able to produce viable pups, suggesting that none of these genes play an important role in fertility.

### 2.6. Characterization of Plpp5, Clptm1l and Itm2c Deficient Mice

RNA-seq data from the Immgen consortium showed that *Plpp5*, *Clptm1l* and *Itm2c* were not highly expressed in hematopoietic stem cells, lymphoid progenitors or developing B cell populations (Figure 1B), making it unlikely that the loss of either protein would impact B cell development and maturation. To ensure that B cell development was normal we analyzed B cell maturation stages in the spleen and bone marrow of each mutant strain. As expected, we did not observe differences in the frequency or number of precursor (B220^+^IgM^−^), immature (B220^lo^IgM^+^), or recirculating mature (B220^hi^IgM^+^) B cells in the bone marrow of WT mice compared with *Plpp5*^−/−^, *Clptm1l*^del/del^ or *Itm2c*^−/−^ mice (Supplementary Figure S1A,B). Furthermore, we did not detect any changes in the frequency or total number or naïve mature (B220^+^IgD^hi^IgM^+^) or immature (B220^+^IgD^lo^IgM^hi^) B cell in the spleens of *Plpp5*, *Clptm1l* or *Itm2c* deficient mice compared to WT mice (Supplementary Figure S1C,D).

**Figure 6:**
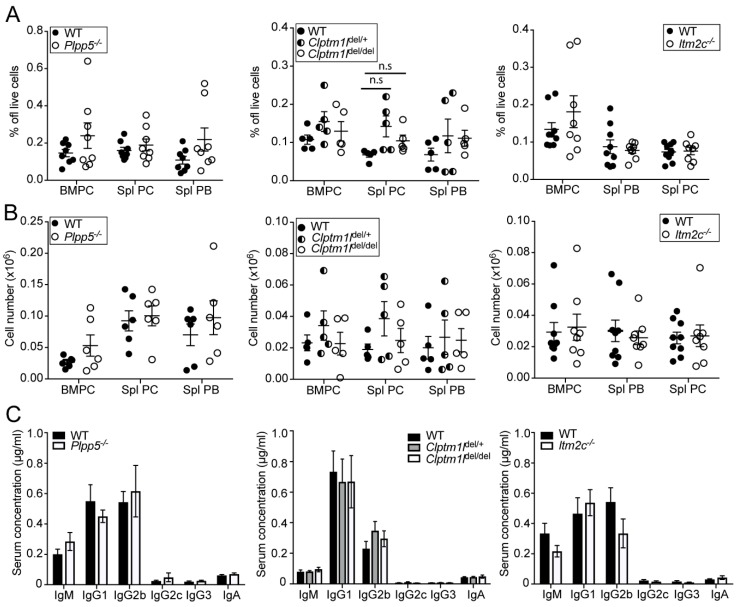
Characterization of ASC populations in *Plpp5*^−/−^, *Clptm1l*^del/+^*, Clptm1l*^del/del^ and *Itm2c*^−/−^ mice. (**A**) Frequency and; (**B**) total cell number of bone marrow plasma cells (BMPC), splenic plasma cells (Spl PC) and splenic plasmablasts (Spl PB) in *Plpp5*^−/−^, *Clptm1l*^del/+^*, Clptm1l*^del/del^, *Itm2c*^−/−^ and age-matched WT mice. Results are combined from two (*Clptm1l*) or three (*Plpp5*, *Itm2c*) independent experiments. Each dot represents a single mouse, horizontal lines show the means ± SEM; (**C**) serum concentration of immunoglobulin isotypes in *Plpp5*^−/−^, *Clptm1l*^del/+^, *Clptm1l*^del/del^, *Itm2c*^−/−^ and age- and sex-matched WT mice as determined by ELISA. Data are the mean of six samples from two independent experiments ± SEM. Statistical significance was analyzed using unpaired *t*-test, correcting for multiple comparisons. n.s., not significant (*p* > 0.05).

To determine whether the loss of *Plpp5*, *Clptm1l* or *Itm2c* influenced the generation of ASCs in vivo, we analyzed the steady state ASC populations in both the BM and spleen in each of the mutant mouse strains. We crossed each strain to Blimp-1-GFP mice [19], which allowed us to differentiate between the PC and the plasmablast (PB) populations in the spleen. We did not observe any marked difference in the frequency of BM PCs (CD138^+^Blimp-1-GFP^+^), Spl PCs (CD138^+^Blimp-1-GFP^hi^) or Spl PBs (CD138^+^Blimp-1-GFP^int^) between WT and the *Plpp5*^−/−^, *Clptm1l*^del/+^, *Clptm1l*^del/del^ or *Itm2c*^−/−^ mice. (Figure 6A,B). We also did not detect a significant difference in the serum concentrations of IgM, IgG1, IgG2b, IgG2c, IgG3 or IgA in the mice deficient in *Plpp5*, *Clptm1l* or *Itm2c* when compared with WT controls (Figure 6C).

To determine whether the loss of Plpp5, Clptm1l or Itm2c would alter the response to stimulation, we isolated naïve B cells from mice of each genotype and cultured them under multiple T cell-dependent (CD40 Ligand (CD40L) + Interleukin (IL)-4 ± IL-5), T cell-independent (LPS) or mixed (LPS + IL-4) stimulation conditions. We did not observe any difference in the differentiation of B cells to ASCs from any of the mutant mice under any of the conditions tested (Supplementary Figure S2A). Additionally, there was no observable defect in the ability of *Plpp5*, *Clptm1l* or *Itm2c* deficient B cells to undergo immunoglobulin class-switch recombination under any of the tested stimulation conditions (Supplementary Figure S2B). Interestingly, we observed a significant increase in the proportion of *Clptm1l*^del/+^ B cells that had undergone class-switch recombination following T cell-dependent stimulation when compared to WT cells. We next used retroviral transduction to ectopically express each gene in activated B cells and found that this premature expression did not impact on the efficiency of ASC differentiation or class-switch recombination in LPS stimulated B cells (Supplementary Figure S2C,D).

**Figure 7:**
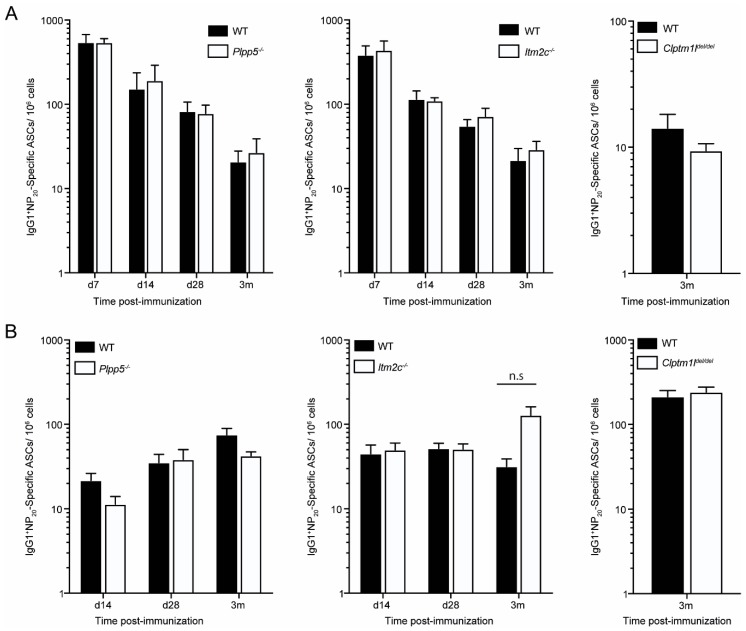
Characterization of the antigen-specific antibody response of *Plpp5*^−/−^ *Clptm1l*^del/del^ and *Itm2c*^−/−^ mice. Mice were immunized intraperitoneally with NP-KLH in alum and, at the indicated time post-immunization, the number of NP20-specific IgG1+ ASCs in the (**A**) spleen and (**B**) BM was determined by ELISpot. *Plpp5* data are combined from three independent experiments at each time point where *n* = 3–6. *Itm2c* data are combined from 1 (day (d) 7) or 2 (d14, d28, 3 months (m)) independent experiments where *n* = 3–4. *Clptm1l* data are from 1 experiment where *n* = 4. Data are the mean ±SEM. Statistical significance was analyzed using unpaired t-test, correcting for multiple comparisons. n.s., not significant (*p* > 0.05).

Finally, we examined the ability of *Plpp5*, *Clptm1l* and *Itm2c* deficient mice to form an antigen specific response following immunization. We immunized *Plpp5*^−/−^, *Clptm1l*^del/del^, *Itm2c*^−/−^ and age-matched WT controls with 4(hydroxy-3-nitrophenyl) acetyl coupled to Keyhole Limpet Hemocyanin (NP-KLH) and determined the frequency of antigen-specific ASCs at multiple timepoints post-immunization (Figure 7). No significant difference was observed in the number of NP-specific ASCs in *Plpp5*^−/−^ and *Itm2c*^−/−^ mice when compared to WT controls at any examined timepoint. Unfortunately, due to the survival disadvantage in the *Clptm1l*^del/del^ mice we were unable to obtain enough age-matched *Clptm1l*^del/del^ mice to do multiple immunization timepoints. However, NP-specific ASCs were detectable in *Clptm1l*^del/del^ mice 3 months post-immunization, indicating that *Clptm1l* deficient mice are capable of mounting a long-lived response to immunization. Together, these data demonstrate that *Plpp5*, *Clptm1l* and *Itm2c* are dispensable for the differentiation of B cells into ASCs, both in vivo and in vitro.

## 3. Discussion

Despite recent improvements in the treatment of MM, it remains an incurable disease. The use of mAb therapies in the treatment of MM has been promising, with recent approvals for anti-SLAMF7 and anti-CD38 mAbs [20,21]. However, the presence of resistant MM cells means that patients often relapse. This highlights the continuing need for novel methods to target MM cells as combination therapies that kill MM cells through multiple approaches increases the likelihood that complete clearance of the cancer can be achieved. Surface molecules present exciting candidates for immunotherapies as they allow for the selective targeting of a cell population either through mAbs or, more recently, through chimeric antigen receptor T cells, which combine the specificity of mAbs with the cytotoxic capabilities of conventional CD8^+^ T cells [20]. Therefore, we chose to focus on surface proteins that are expressed in ASCs and consequently MM. In this study we have examined three proteins, PLPP5, CLPTM1L and ITM2C, which are ASC surface proteins that we believe present promising candidates for an ASC-directed immunotherapy, but whose biological functions were largely unknown.

PLPP5 (also known as PPAPDC1B and HTPAP) encodes a lipid phosphatase that has been shown to be present on the plasma membrane and within the cytoplasm of PLPP5 overexpressing human hepatocellular carcinoma cell lines [22,23]. Although the function of PLPP5 has not been determined, it has been proposed to have an oncogenic role in breast cancer. *PLPP5* is estimated to be amplified in 10–15% of ductal breast carcinomas, and its knock-down in breast cancer cell lines causes an increase in apoptosis [24,25]. Similar observations have also been reported in pancreatic adenocarcinoma and small-cell lung cancer cell lines [25]. Within the mouse immune system we found that *Plpp5* expression was highly restricted to ASCs. Although its expression in ASCs and MM cell was conserved in human samples, we also observed considerable *PLPP5* expression in naïve, but not germinal center or memory B cells, suggesting that any mAb therapy based on PLPP5-binding and depletion would also target naïve B cells. As B cell depletion using Rituximab has been successfully used for many years in lymphoma [26] and some autoimmune contexts [27], the expression of PLPP5 in B cells is not likely to be an insurmountable obstacle for an anti-PLPP5-based therapy for MM or Ab mediated autoimmunity.

CLPTM1L is a close homologue of Cleft lip and palate transmembrane protein 1 (CLPTM1) and is predicted to contain six transmembrane domains [17]. There is conflicting data regarding the subcellular localization of CLPTM1L, with previous studies observing it exclusively in the mitochondria [28] or endoplasmic reticulum [29], while the data presented here and another recent study [17], have found evidence for localization to the plasma membrane. It is possible that the location of CLPTM1L is dependent on cell context or that aberrant localization is a result of the high levels of expression seen in the examined cancer cell lines. Mutations in *CLPTM1L* have been identified as risk factors in a range of cancers, including lung [30,31], pancreatic [32], colorectal [33], glioma [34] and testicular germ cell cancer [35], and the expression of *CLPTM1L* is significantly increased in cancerous cells when compared to healthy adjacent tissues [29,30,36]. This increase in expression in malignant cells was also evident in our analysis of primary MM samples compared with plasma cells. The knock-down of *CLPTM1L* with siRNA or targeting with an anti-CLPTM1L antibody increased the sensitivity of *CLPTM1L* overexpressing cancer cells to killing through genotoxic stress inducing agents [17,30]. Together, these observations suggest that targeting CLPTM1L in MM could potentially increase sensitivity to other treatments, however, this remains to be tested.

Curiously, we observed that *Clptm1l*^del/del^ pups appeared to not only be born at lower than anticipated frequencies, but to have an initial survival disadvantage compared to their *Clptm1l*^+/+^ and *Clptm1l*^del/+^ littermates. *Clptm1l*^del/del^ mice that survived past two days old showed no sign of physical defect or fitness disadvantage, suggesting that Clptm1l plays an important role in early life but is dispensable later on. Future investigation into the role of Clptm1l in adult mice should utilize the conditional strain that we have generated to avoid the complication of the poor neonatal survival that we have observed.

ITM2C is a member of the type 2 integral transmembrane family that has previously been reported to be localized to the plasma membrane [37], Golgi apparatus [38] and within lysosomes [39]. Previous work has focused on its role within the brain, as it is highly expressed in both the embryonic and adult mouse brain, as well as in adult human brain tissue [40,41]. Within the immune system, we observed strong expression of ITM2C in mouse and human ASCs, but also expression in dendritic cells and some macrophage populations. Whether these non-ASC expression domains will impact on the utility of any anti-ITM2C mAb therapy remains to be determined, but it is noteworthy that the two clinically approved mAb for MM, Daratumumab (CD38) and Elotuzumab (SLAMF7) target antigens expressed on ASC and multiple other cell types.

ITM2C is thought to have a role in the inhibition of the β-amyloid protein processing pathway based on the observations that it is capable of directly binding the β-amyloid precursor protein and *Itm2c* expression is inversely correlated with β-amyloid peptide production [38,42]. Due to the high expression of *Itm2c* within the embryonic mouse brain we were surprised to find that *Itm2c*^−/−^ pups were viable and showed no evidence of a fitness disadvantage when compared to littermates. Additionally, *Itm2c* is expressed in the testis during sexual maturation [43], however, *Itm2c*^−/−^ male mice were able to produce pups indicating that the loss of *Itm2c* does not influence fertility.

Despite the increase in expression of *Plpp5*, *Clptm1l* and *Itm2c* during B cell differentiation, we did not identify any of these genes as being essential for either the generation of ASCs in steady state, either in vitro or in vivo, or for the ability of ASCs to secrete antibody. The presence of long-lived plasma cells in the spleen and bone marrow of all three KO mice suggests that these genes are also not required for the long-term survival of ASCs, or for homing to and retention within the bone marrow. This conclusion is supported by the presence of normal numbers of antigen-specific ASC after a T-dependent immunization. Whether this is due to functional redundancy with related proteins is at present unclear. However, it is noteworthy that the ITM2 family consists of three members (a, b, c) that show around 40% amino acid identity [40] and similar expression domains in late B cells (www.immgen.org) raising the possibility of redundancy between ITM2 family members.

This work describes the first investigation into the roles of PLPP5, CLPTM1L and ITM2C within the immune system. Furthermore, this work details the first time mutant mouse strains of each gene have been described and presents them as valuable tools for the investigation into the roles of these genes within other cell populations of interest, particularly in the context of cancer. With their surface localization and their high expression within both ASCs and MM samples, PLPP5. CLPTM1L and ITM2C represent enticing candidates for novel immunotherapies to target these cell populations. Adult mutant mice showed no apparent fitness defect or disease phenotype, which suggests that targeting these molecules will have minimal on target side effects. The next stage in the investigation of these proteins will be the generation of mAbs, first to allow for further investigation into the biology of PLPP5. CLPTM1L and ITM2C and then to determine their potential for therapeutic use.

## 4. Materials and Methods

### 4.1. Mice

Mice were bred and maintained on a C57BL/6 background and housed in a specific pathogen free facility. *Plpp5* mutant mice were generated using *Plpp5*^tm1(KOMP)Mbp^ embryonic stem (ES) cells obtained from the KOMP Repository. *Clptm1l* mice were made using Clptm1l^tm1a^ (^EUCOMM)Hmgu^ ES cells obtained from the EUCOMM Consortium. In the case of *Itm2c*, we originally obtained targeted ES cells from EUCOMM (ID:69805, clones EDP0351_4_B07 and _F05). No germline transmission occurred with the B07 ES cells, while the F05 ES cells contained a gene duplication at the *Itm2c* locus and were thus not useful for to generate a loss-of-function allele. As an alternative, *Itm2*c mice were generated in house using CRISPR/Cas9. Guide sequences were AACTGCTAAAGAGGGTGGTC and GGTCGACATTCACTATAGTC. In the first generation, mice were generated that bore *Itm2c* deletions with micro heterogeneities around the deletion endpoints. As founders, we chose to mate a male and female that carried identical mutated sequences. Primers for genotyping were as follows: Plpp5WT-F (GTCTTAGTGTTGGCAAGTAGCTATGGG), Plpp5WT-R (CCATCTGCTTGG AGAAGAGTAAGCC), Plpp5KO-F (GCCTGTCAATCTTCCCCGTTTCCTCCCC), Plpp5KO-R (GGTGAGAGGAGAATTCTGGAATCCATCC), ClptWT-F (TCCTATTCATCACCCTGTGCCAGG), ClptWT-R (CCCACCTCTGTTAGAGCCTCAGACTAC), ClptDel-F (TCCTATTCATCACCCTGTGC CAGG), ClptDel-R (CTGATGGCGAGCTCAGACCATAACTTCG), Itm2cWT-F (AAATTCGGGCTG ATTGTTTG), Itm2cWT-R (CCTAAGAGCTCCTGGTGACG), Itm2cKO-F (TTCCCATGAACTCCTT GGTC), Itm2cKO-R (GCGAGGCAAGTGAGGTAGAC). Blimp-1-GFP [19], Flp [44] and Cre [45] recombinase expressing mice have been described previously. All animal experiments were conducted in accordance with protocols approved by the Walter and Eliza Hall Institute Animal Ethics Committee (2016.002, approved 31 March 2016).

### 4.2. Bioinformatic Analysis

RNAseq data for mouse immune cells derives from (GSE60927) [9] and the Immunological Genome Consortium database (www.immgen.org). RNA-seq from human hematopoietic cell populations derives from the BLUEPRINT consortium (www.blueprint-epigenome.eu) and Fedele et al. (unpublished). Pairwise sequence alignments between the mouse and human protein sequences were performed using EMBOSS Needle [46]. In silico analysis of the protein structures used the Phobius [13], Spoctopus [15], TMHMM2 [14] and TMMOD [12] tools.

### 4.3. Generation, Expression and Detection of Tagged Proteins

The coding sequence of the predominant isoform of human *CLPTM1L* (NP_110409) and *PLPP5* (NP_001096029) were synthesized at GeneArt (Thermo Fisher, Waltham, MA, USA) inframe with a 5′ FLAG-tag coding sequence (DYKDDDDK) and 3′ a HA-tag coding-sequence (YPYDVPDYA). *ITM2C* (NP_071862) coding sequence was synthesized with a 3′ FLAG-tag coding sequence. The coding sequences were cloned into a pCDNA 3.1-based expression vector and transiently transfected into FreeStyle 293-F (FS-293F) using 293fectin (Gibco, Thermo Fisher, Waltham, MA, USA) according to manufacturer’s protocol. Expression was enhanced by adding Lucratone Lupin (Millipore-Sigma, Burlington, MA, USA) at 0.5% *v*/*v* final concentration 18 h post-transfection. FS293-S cells were cultured in Freestyle 293 Expression Media (Gibco). Cells were analyzed 48 h post-transfection.

For intra-cellular staining, cells were fixed and permeabilized using BDCytofix/Cytoperm system according to the manufacturer’s guidelines (BD Biosciences, Franklin Lakes, NJ, USA). Cells were blocked with FcR Blocking reagent (130-059-901, Miltenyi Biotec, Bergisch Gladbach, Germany) and stained using the following antibodies: Mouse anti-HA-Alexa Fluro-488 mAb (2350, Cell Signaling Technology, Danvers, MA, USA), Rabbit anti-FLAG polyclonal (p)Ab (2368, Cell Signaling Technology), Rabbit anti-Human-CLPTM1L pAb (GTX116893, Genetex, Irvine, CA, USA), Rabbit anti Human-ITM2C pAb (GTX116904, Genetex) and Rabbit anti-Human-PLPP5 (LS-B4624, LifeSpan BioSciences, Seattle, WA, USA). Rabbit IgG isotype control (GTX35035, Genetex) was used in some experiments. Unconjugated rabbit pAb were detected with secondary antibody anti-Rabbit IgG Alexa Flour 647 (4414, Cell Signaling Technology). Cells were analyzed on FACSCanto (BD Biosciences).

### 4.4. Patient Samples

BM from MM patients (newly diagnosed, relapsed/refractory) or healthy donors was obtained following written informed consent as per Alfred Hospital Human Ethics Committee-approved protocol. Isolation of bone marrow mononuclear cells (BMMNC), determination of MM cell proportion and isolation of CD138^+^ cells from MM cells and plasma cells from healthy donors was performed as previously described [47]. Briefly, Ficoll Plaque Plus (GE Healthcare, Chicago, IL, USA) was utilized to isolate BMMNC as per manufacturer’s guidelines. Red blood cells were removed using red blood cell lysis buffer (10 mmol/L KHCO_3_, 150 mmol/L NH_4_Cl and 0.1 mmol/L EDTA, pH 8.0) for 5 min at 37 °C followed by washing with sterile phosphate buffered saline (PBS). The proportion of MM or normal plasma cells (CD38^+^CD45^−^CD138^+^) in BMMNC isolated from each patient was determined through flow cytometric enumeration on a FACSCalibur Flow Cytometer (BD Biosciences). To isolate MM cells, anti-CD138 MACS beads were employed using manufacturer’s guidelines (Miltenyi Biotec). CD138^+^ cells were selected through magnetic isolation using an MS-column (Miltenyi Biotec). For normal BM, flow cytometry was performed to ensure that proportion of plasma cells fell within the normal range (<3%). Normal plasma cells were isolated through the utilization of plasma cell isolation kit II (Miltenyi Biotec). Purified cells were stored in TRIzol (Life Technologies, Thermo Fisher, Waltham, MA, USA). Human B cells and T cell controls were purified from peripheral blood mononuclear cells (PBMCs) derived from buffy coats using anti-CD19-MACS beads or anti-CD3-MACS beads respectively. All samples were de-identified for this study.

Protocols were approved by the Alfred Hospital Human Ethics Committee, Monash Health and the Walter and Eliza Hall Institute Human Research Ethics Committees.

### 4.5. Flow Cytometry

#### 4.5.1. Mouse

Single cell suspensions were labeled with the following mAbs: anti-CD138 (281-2), anti-B220 (RA3-6B2), anti-IgG1 (X56), all from BD Biosciences; anti-IgD (11–26c.2a) and anti-IgM (II/41), both from eBioScience (Affymetrix, Santa Clara, CA, USA). Cells were analyzed on FACSCanto Flow Cytometer (BD Biosciences).

#### 4.5.2. Human

Human multiple myeloma cell lines were labeled with Rabbit anti-Human-PLPP5 pAb (LS-B4624, LifeSpan BioSciences) or Rabbit IgG isotype control (GTX35035, Genetex). Unconjugated pAb was detected with secondary antibody anti-Rabbit IgG Alexa Flour 647 (4414, Cell Signaling Technology). Cells were analyzed on FACSCanto (BD Biosciences).

### 4.6. Multiple Myeloma Cell Lines

Human multiple myeloma cell lines were grown in RPMI supplemented with 10% FCS, 1% l-Glutamine, 1% HEPES, 1% non-essential amino acids, 1% Sodium pyruvate, 50 μM β-mercaptoethanol. Recombinant Human IL-6 (R&D Systems, Minneapolis, MN, USA) was supplied when necessary. 1 × 10^6^ cells were harvested in experimental triplicates or duplicates over subsequent passages for RNA purification.

### 4.7. Immunohistochemistry

Paraffin-embedded (de-identified) archival samples were de-waxed, and antigen retrieval was performed (120 C under 20 psi pressure, 3 min, 1 mM EDTA, pH 9.0). Blocking was with 5% FCS in PBS, 15 min. Primary rabbit anti-CLPTM1L antibody (HPA014791, Sigma-Aldrich, St. Louis, MO, USA) was added at 1:100, incubated at RT for 15 min, washed with 5% FCS in PBS, then the secondary antibody (goat anti-rabbit Ig-HRP, sc-2004, 1:500; Santa Cruz Biotechnology, Dallas, TX, USA) was added and incubated for 40 min, washed and developed using standard protocols.

### 4.8. B Cell Isolation and Cell Culture

Naïve splenic B cells were isolated using a B Cell Isolation Kit (Miltenyi Biotech,) and cultured in RPMI (supplemented with 10% FCS, 1% l-Glutamine, 1% HEPES, 1% non-essential amino acids, 1% Sodium pyruvate, 50 μM β-mercaptoethanol) with combinations of 20 µg/mL lipopolysaccharide (LPS, Sigma-Aldrich) and 100 ng/mL CD40 ligand (CD40L), 10 ng/mL Interleukin-4 (IL-4) and 5 ng/mL Interleukin-5 (IL-5) (all from R&D Systems).

### 4.9. RNA Isolation and qRT-PCR

#### 4.9.1. Mouse

Naïve B cells were culture for 4 days in the presence of LPS before being resuspended in RLT lysis buffer (QIAGEN, Venlo, Netherlands) and transferred to a QIA shredder column (QIAGEN) for lysis and homogenization. RNA extraction was performed using an RNeasy Plus Mini Kit (QIAGEN). cDNA was generated using iScript reverse transcription supermix (BioRad, Hercules, CA, USA). qPCR was performed using Taqman probes (*Hprt*: Mm00446968_m1, *Clptm1l*: Mm00524746_m1, *Plpp5*: Mm01210970_m1, *Itm2c*: 00499081_m1) and TaqMan Universal Mastermix all from Applied Biosystems.

#### 4.9.2. Human

Primary cells and myeloma cell lines were homogenized in RLT lysis buffer using the QIA shredder columns (QIAGEN). RNA extraction for myeloma cell lines and PBMC derived control B and T cells was performed using the RNeasy mini kit (QIAGEN) including a genomic DNA digestion step. RNA extraction from primary human plasma cells and MM cells was performed using a RNeasy micro kit (QIAGEN). cDNA was generated using SSIII First-Strand cDNA synthesis kit (Invitrogen, Thermo Fisher Scientific, Waltham, MA, USA). qPCR used Taqman probes (Applied Biosystems, Foster City, CA, USA): *CLPTM1L*: Hs00363947_m1, *ITM2C*: Hs00985194_g1, *PLPP5*: Hs00998335_g1, *SLAMF7*: HS00221793_m1, *GPBP1*: Hs00607556_m1, *GAPDH*: Hs02758991_g1, *TPT1*: Hs01044518_g1. Raw qPCR data was imported into LinReg [48] software to determine Cq and reaction specificities. Final analysis was conducted in qBASEplus (Biogazelle, Zwijnaarde, Belgium).

### 4.10. ELISA

Blood for serum antibody analysis was collected from all mice at 49 days of age. Plates were coated with anti-mouse IgM, IgG1, IgG2b, IgG2c, IgG3 or IgA (Southern Biotech, Birmingham, AL, USA) for 24 h before the addition of diluted serum. IgM, IgG1, IgG2b, IgG3 standards were obtained from Sigma-Aldrich, IgG2c standard was from Southern Biotech, and IgA standard was from Organon Tekcika–Cappel (Durham, NC, USA). Serum Ig was detected with anti-IgM-HRP, anti-IgG1-HRP, anti-IgG2b-HRP, anti-IgG2c-HRP, anti-IgG3-HRP, anti-IgA-biotin and streptavidin-HRP (Southern Biotech) and visualised using ABTS substrate (2,2′-Azinobis (3-ethylbenzthiazoline Sulfonic Acid); Sigma-Aldrich). All samples and standards were measured in duplicate.

### 4.11. Retroviral Transduction of B Cells

Plasmids containing pMD1-gag-pol, pCAG-Eco, and pMIG (expressing either GFP alone (empty vector) or murine *Plpp5*, *Itm2c* or *Clptm1l* and GFP) were transfected into 293T cells using the Calcium phosphate method. Retroviral supernatant was collected after 48 h and transferred to B cells that had been pre-stimulated with LPS for 24 h. Transduction was performed using a spin infection in the presence of polybrene (4 µg/mL). Transduced cells were then cultured in LPS for a further 3 days before analysis.

### 4.12. Immunization

4(hydroxy-3-nitrophenyl) acetyl coupled to Keyhole Limpet Hemocyanin (NP-KLH) (Biosearch Technologies, Petaluma, CA, USA) at concentration of 1 mg/mL was added to Imject Alum (Thermo Scientific) at a ratio of 1:1. 200 μL was injected into mice intraperitoneally. All mice were at least 7 weeks old at the time of immunization.

### 4.13. ELISpot

Multiscreen HA plates (Millipore-Sigma) were coated with 10 µg/mL NP20-BSA for 4 h before the addition of cells. Plates were then incubated for 14–18 h at 37 °C 10% CO_2_. NP-specific antibody was detected with anti-IgG1-HRP and visualized using 3-amino-9-ethylCarbazole (Sigma-Aldrich).

### 4.14. Statistical Analysis

Gene expression in human MM, plasma cells and B cells were analyzed using one-way ANOVA with Tukey’s multiple comparison test on log transformed data. Data from *Plpp5*^−/−^ *Itm2c*^−/−^ and *Clptm1l*^del/del^ experiments were analyzed using unpaired t-tests using the Holm–Sidak method to correct for multiple comparisons.

## Figures and Tables

**Table 1:** Genotypes of offspring from *Clptm1l*^del/+^ with *Clptm1l*^del/+^ mating.

Genotype:	*Clptm1l* ^+/+^	*Clptm1l* ^del/+^	*Clptm1l* ^del/del^
Expected	13.25	26.5	13.25
Observed	21	29	3 *

* *p* = 0.00175.

**Table 2:** Survival of *Clptm1l*^del/del^ pups.

Total Number Born	Number Survived Past Day 2
37	5

## Supplementary Materials

Supplementary materials can be found at http://www.mdpi.com/1422-0067/19/8/2161/s1.
