# Supplementary material for: Mining the Plasma Cell Transcriptome for Novel Cell Surface Proteins
Source: Int J Mol Sci. 2018 Jul 24;19(8):2161. doi: 10.3390/ijms19082161 (PMC6121261; doi:10.3390/ijms19082161)
Supplement: Supplementary file 1 [file ijms-19-02161-s001.zip › ijms-328274-SI.pdf]

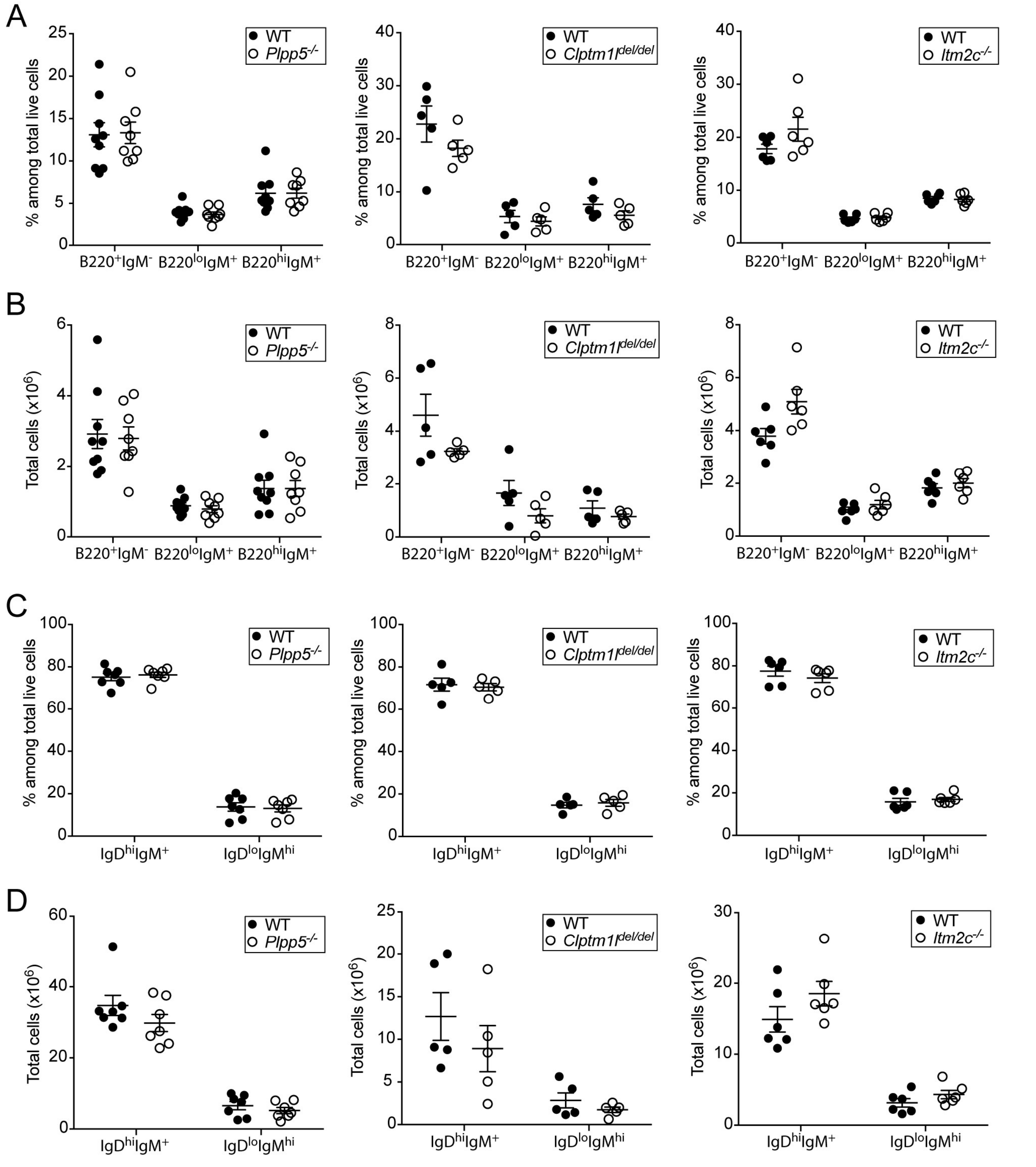

Supplementary Figure 1. *Plpp5*$^{-/-}$, *Clptm1l*$^{del/del}$ and *Itm2c*$^{-/-}$ mice have normal B cell development and maturation. (**A**) Frequency and; (**B**) Number of precursor (B220^{+}IgM^{-}), immature (B220^{lo}IgM^{+}) an recirculating (B220^{+}IgM^{hi}) B cells in the bone marrow of *Plpp5*$^{-/-}$, *Clptm1l*$^{del/del}$, *Itm2c*$^{-/-}$ and age-matched WT mice. (**C**) Frequency and; (**D**) Number of mature (IgD^{hi}IgM^{+}) and immature (IgD^{lo}IgM^{hi}) B cells in the spleen of *Plpp5*$^{-/-}$, *Clptm1l*$^{del/del}$, *Itm2c*$^{-/-}$ and age-matched WT mice. Results are combined from 2 (*Clptm1l*) or 3 (*Plpp5*, *Itm2c*) independent experiments. Horizontal line shows the mean ±SEM. Each dot represents an individual mouse.

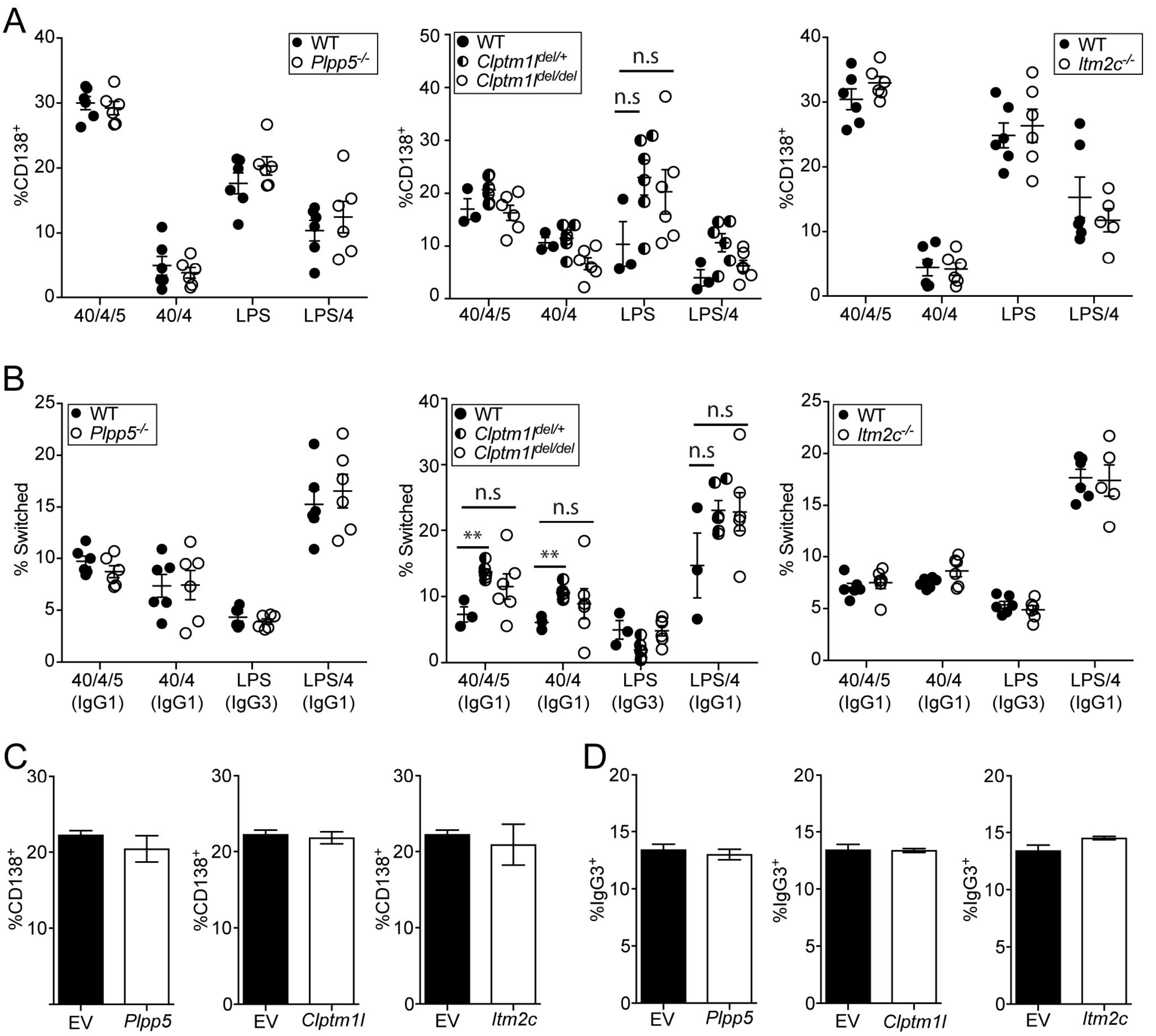

Supplementary Figure 2. *Plpp5*$^{-/-}$, *Clptm1l*$^{del/del}$ and *Itm2c*$^{-/-}$ B cells respond normally to *in vitro* stimulation. (**A-B**) Naïve splenic B cells were isolated from *Plpp5*$^{-/-}$, *Clptm1l*$^{del/+}$, *Clptm1l*$^{del/del}$, *Itm2c*$^{-/-}$ and age-matched WT mice and cultured for 4 days in CD40L, IL-4, IL-5 (40/4/5), CD40L, IL-4 (40/4), LPS or LPS/IL-4 (LPS/4) before analysis by flow cytometry. The proportion of B cells that had undergone (**A**) differentiation to ASCs (CD138^{+}) or (**B**) Immunoglobulin class-switch recombination is shown. Each dot represents an individual mouse. (**C-D**) Naïve splenic B cells were isolated and stimulated for 24 hours with LPS before retroviral transduction with full length Plpp5, Clptm1l, Itm2c or empty vector (EV) control. After a further 3 days of LPS stimulation, cells were analyzed by flow cytometry for the proportion of transduced (GFP^{+}) cells that had undergone (**C**) ASC differentiation (CD138^{+}) and (**D**) Immunoglobulin class-switch recombination. Results are the mean ±SEM from 2 independent experiments. Statistical significance was analyzed using unpaired t-test, correcting for multiple comparisons. ** $P<0.01$ for the indicated comparison. n.s, not significant ($P>0.05$).
